# Assessment of depression and anxiety in a cohort of heart transplantation recipients

**DOI:** 10.1192/j.eurpsy.2026.11033

**Published:** 2026-07-31

**Authors:** R. Sánchez-González, E. Monteagudo-Gimeno, L. Pintor-Pérez

**Affiliations:** 1 Department of Psychiatry, Institut de Salut Mental, Centre Emili Mira. Hospital del Mar, Barcelona; 2 Department of Psychiatry, Benito Menni Mental Health Care Complex, Sant Boi de Llobregat; 3 Consultation-Liaison Service. Department of Psychiatry, Institut de Neurociències. Hospital Clínic i Provincial de Barcelona., Barcelona, Spain

## Abstract

**Introduction:**

Heart transplantation (HT) constitutes a potentially life-saving intervention for patients with end-stage cardiac disease. Nonetheless, it remains one of the most invasive and psychologically demanding procedures available. Studies report psychiatric morbidity rates of approximately 50% among individuals undergoing HT, predominantly characterized by depressive and anxiety disorders. Although these rates tend to decline during the postoperative period, approximately one-third of HT recipients continue to experience psychological complications in the long-term course.

**Objectives:**

To assess depression and anxiety using the Hospital Anxiety and Depression Scale (HADS) in a cohort of patients followed for one year after HT.

**Methods:**

An assessment was conducted on 37 adult patients enrolled in the HT program at the Hospital Clinic of Barcelona between 2016 and 2023. During the waiting list phase, the following variables were analyzed: age, sex, duration of disease progression, etiology of cardiopathy, Axis I disorders diagnosed using the Structured Clinical Interview for DSM-IV, HADS scores, and details of psychopharmacological treatment. Additionally, HADS assessments were repeated one year post-HT.

**Results:**

**Demographic and clinical characteristics:** The sample comprised 37 patients with a mean age of 53.65 ± 9.77 years. Among them, 26 were male (70.3%) and 11 female (29.7%). The average duration of cardiac disease progression was 11.11 ± 8.15 years. Ischemic cardiomyopathy was present in 54.1% of patients, while 21.6% had non-ischemic cardiomyopathy. The clinical characteristics of this cohort are consistent with the general patient profile typically enrolled in a heart transplantation program.

**Psychiatric evaluation:** Axis I psychiatric diagnoses were identified in 27% of the sample (10 patients), encompassing depressive, anxiety, sleep, and adjustment disorders. All diagnosed patients were receiving psychopharmacological treatment at the time of inclusion on the waiting list. T-test showed statistically significant differences on the three scores of HADS between the two assessments:

Waiting list period: the mean score of HADS was 10.68 (SD = 5.51), with a mean score of 6.08 (SD = 3.5) in anxiety subscale and 4.59 (SD = 2.83) in depression subscale.

12-month follow-up : the mean score of HADS was 7.73 (SD = 5.28), with a mean score of 4.84 (SD = 3.32) in anxiety subscale and 2.89 (SD = 2.59) in depression subscale.

**Table 1.** Table 1. Long description.

	Waiting list period	12-Month Follow-Up	t	p
**HADS total**	10,68 (5,51)	7,73 (5,28)	3,428	0,002
**HADS depression**	4,59 (2,83)	2,89 (2,59)	4,118	0,001
**HADS anxiety**	6,08 (3,5)	4,84 (3,32)	2,166	0,037

**Conclusions:**

Although the HADS scores in our sample were below the established cut-off points for screening psychiatric morbidity (<12 for the total score and <8 for the subscales), a significant reduction in anxiety and depression symptoms was observed 12 months after HT.

**Disclosure of Interest:**

None Declared

## Long descriptions

### Table 1. Long description

The table consists of five columns: Category, Waiting list period, 12-Month Follow-Up, t-value, and p-value. Data is presented as mean with standard deviation in parentheses.

* H A D S total: Waiting list period 10.68 (5.51), 12-Month Follow-Up 7.73 (5.28), t = 3.428, p = 0.002.

* H A D S depression: Waiting list period 4.59 (2.83), 12-Month Follow-Up 2.89 (2.59), t = 4.118, p = 0.001.

* H A D S anxiety: Waiting list period 6.08 (3.5), 12-Month Follow-Up 4.84 (3.32), t = 2.166, p = 0.037.

Navigate back to Table 1..

